# Discrimination of Gain Increments in Speech

**DOI:** 10.1177/2331216519886684

**Published:** 2019-11-18

**Authors:** Benjamin Caswell-Midwinter, William M. Whitmer

**Affiliations:** 1 Division of Clinical Neuroscience, Faculty of Medicine and Health Sciences, University of Nottingham, UK; 2 College of Medical, Veterinary, and Life Sciences, University of Glasgow, UK

**Keywords:** just-noticeable differences, hearing aids, hearing-aid gain, hearing-aid fitting

## Abstract

During a hearing-aid fitting, the gain applied across frequencies is often adjusted from an initial prescription in order to meet individual needs and preferences. These gain adjustments in one or more frequency bands are commonly verified using speech in quiet (e.g., the clinician’s own voice). Such adjustments may be unreliable and inefficient if they are not discriminable. To examine what adjustments are discriminable when made to speech, this study measured the just-noticeable differences (JNDs) for gain increments in male, single-talker sentences. Sentences were presented with prescribed gains to the better ears of 41 hearing-impaired listeners. JNDs were measured at *d’* of 1 for octave-band, dual-octave-band, and broadband increments using a fixed-level, same-different task. The JNDs and interquartile ranges for 0.25, 1, and 4 kHz octave-band increments were 6.3 [4.0–7.8], 6.7 [4.6–9.1], and 9.6 [7.3–12.4] dB, respectively. The JNDs and interquartile ranges for low-, mid-, and high-frequency dual-octave-band increments were 3.7 [2.5–4.6], 3.8 [2.9–4.7], and 6.8 [4.7–9.1] dB, respectively. The JND for broadband increments was 2.0 [1.5–2.7] dB. High-frequency dual-octave-band JNDs were positively correlated with high-frequency pure-tone thresholds and sensation levels, suggesting an effect of audibility for this condition. All other JNDs were independent of pure-tone threshold and sensation level. JNDs were independent of age and hearing-aid experience. These results suggest using large initial adjustments when using short sentences in a hearing-aid fitting to ensure patient focus, followed by smaller subsequent adjustments, if necessary, to ensure audibility, comfort, and stability.

## Introduction

The adjustment of frequency-dependent gain is common in hearing-aid fittings, whether in real-ear verification or in fine-tuning to patient feedback. While fine-tuning to patient feedback is routine (Anderson, Arehart, & Souza, 2018), evidence suggests it is of little objective or subjective benefit (Cunningham, Williams, & Goldsmith, 2001; Saunders, Lewis, & Forsline, 2009). It may be that most electroacoustical adjustments are simply not noticeable, resulting in unreliable patient feedback and inefficient device comparisons. Furthermore, the perceived value of the intervention may be depreciated if the average patient’s expectation of a noticeable difference is not met (Demorest, 1984; McShefferty, Whitmer, & Akeroyd, 2015, 2016).Data proposing benchmarks of noticeable frequency–gain response adjustments would help clinicians judge whether an adjustment should be instantly noticeable to the patient.

The authors previously reported the just-noticeable differences (JNDs) for gain increments in frequency-specific speech–spectrum-shaped noise regions: approximately 3 dB for octave bands from 500  to 4000 Hz (Caswell-Midwinter & Whitmer, 2019). Those data extend the field of profile analysis (Green, 1988) to provide perceptual evidence for baseline gain adjustments from prescription and tolerances for adjusting real-ear gain to prescribed targets. However, those JNDs were only for noise stimuli and octave-wide adjustments; fine-tuning usually has the patient compare gain adjustments made to live voice across multiple frequency bands (Anderson et al., 2018; Dillon, 2012; Jenstad, Van Tasell, & Ewert, 2003; Thielemans, Pans, Chenault, & Anteunis, 2017). Given this, there is a need to examine what gain adjustments are noticeable when made to speech.

Increasingly popular self-fitting technology allows patients to adjust their own devices to real-world stimuli in their own acoustical environments (Keidser & Convery, 2016) rather than in a quiet and unrealistic clinic. This alleviates the need for the patient to recall troublesome situations, and the need for the clinician to interpret patient feedback into adjustments which meet the patient’s subjective criteria. Laboratory studies simulating a variety of acoustical scenarios suggest that both discrete and continuous self-adjustments can provide an efficient and reliable means of meeting patient preference (Boothroyd & Mackersie, 2017; Nelson, Perry, Gregan, & VanTasell, 2018). However, more research is required on the implementation and outcomes of patient-led adjustments given that there is evidence of them being less beneficial, in subjective and objective domains, than clinician-led adjustments (Boymans & Dreschler, 2012). The growing availability of this technology centered on instant comparisons of processed speech furthers the need to examine what gain adjustments are noticeable when made to speech.

Previous research only provides evidence on noticeable broadband gain adjustments. In examining gain preferences with speech in noise, Dirks, Ahlstrom, and Noffsinger, (1993) and Jenstad et al. (2007) reported that adjustments of 4 (for +3 dB signal-to-noise ratio) and 4.5 dB, respectively, resulted in differences in sound quality and intelligibility, while Byrne and Dillon (1986) briefly reported that a 3-dB adjustment resulted in different ratings of intelligibility and pleasantness. Participants in these studies made preference judgments rather than discrimination judgments. Preference is subjective and can be influenced or defined by criteria such as sound quality and intelligibility (as in the aforementioned studies). Discrimination based on a same-different procedure accommodates any internal criteria, whether based on timbre, loudness, clarity, or otherwise, into a basic psychophysical query of whether the stimuli are equal or not (Macmillan & Creelman, 2005). While establishing a patient’s gain preference is vital for a hearing-aid fitting, preference judgments based on differences below the threshold of discriminability can be meaningless and waste valuable clinical time. More importantly for hearing-aid gain, these studies only reported broadband gain adjustments; frequency-specific gain adjustments are central to fine-tuning practices (Jenstad et al., 2003; Sabin, Hardies, Marrone, & Dhar, 2011; Thielemans et al., 2017). It is not known what magnitudes of frequency-specific adjustments are noticeable when made to speech or whether there is an effect of center frequency.

This study directly measured the JNDs for gain increments in speech. Participants listened to sentences that were processed with prescribed National Acoustics Laboratories-Revised (NAL-R) gains and discriminated level increments in a fixed-level (method of constant stimuli), same-different task. Octave-band gain adjustments are often used in real-ear measurements for meeting prescription targets, while larger, multiple octave-band gain adjustments are often used for fine-tuning and automated fittings (Nelson et al., 2018; Sabin et al., 2011). Therefore, both octave-band and dual-octave-band JNDs were measured.

## Methods

### Participants

**Figure 1.**
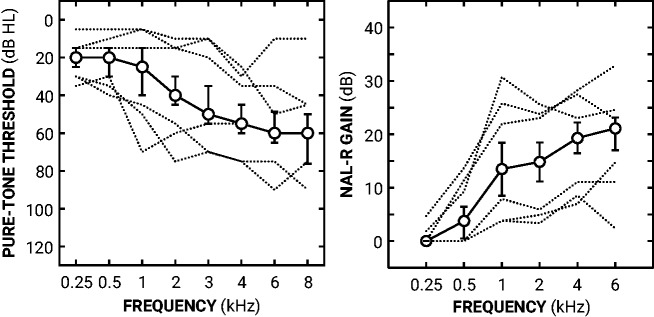
Median audiogram; NAL-R prescribed gains. Left panel shows median pure-tone thresholds across all 41 participants as a function of frequency. Error bars show interquartile ranges (25%–75%). The dotted lines show the better-ear thresholds of participants with the three lowest and three highest BE4FA thresholds. Right panel shows median National Acoustic Laboratory-Revised (NAL-R) prescribed gains across all participants as a function of frequency. Error bars show interquartile range. The dotted lines show the prescriptions of the participants with the three lowest and highest BE4FA thresholds. BE4FA = better ear four-frequency pure-tone threshold average.

Forty-one participants (17 females) were recruited from local audiology clinics. The median age of participants was 69 years (range: 51–75 years). All participants had some degree of hearing loss in at least one ear. All participants had experience wearing hearing aids. The median hearing-aid experience was 10 years (range: 2–35 years), and the median proportion of life wearing hearing aids was 0.14 (range: 0.03–0.53 of life). Unmasked pure-tone thresholds were manually measured for both ears just prior to the experiment (British Society of Audiology, 2011). For the experiment, participants were tested in their better ear (BE), determined by the lower four-frequency pure-tone threshold average (4FA) of their ears. BE4FAs were calculated by averaging pure-tone thresholds at 500, 1000, 2000, and 4000 Hz. Figure 1 shows the median BE audiogram across all 41 participants and key characteristics of the audiogram distributions. The median BE4FA was 35 dB HL (range: 13–59 dB HL). One participant had a conductive element to their hearing loss based on the differences between air and bone conduction thresholds exceeding 20 dB when averaged over three of the five frequencies at 0.5, 1, 2, 3, and 4 kHz (British Academy of Audiology, 2016).

The experiment was ethically approved by the University of Glasgow research ethics system committee (application number 200160138). All participants provided written informed consent prior to the experiment.

### Stimuli

Stimuli were sentences taken from the Bamford–Kowal–Bench (BKB) corpus (Bench, Kowal, & Bamford, 1979), spoken by a native British English male talker. BKB sentences are declarative and mostly consist of five words (e.g., “she cut with her knife”). The sampling rate was 44.1 kHz, but the spectrum of the original corpus was limited with a steep 10-kHz low-pass filter. The average sentence duration was 1634 ms, ranging from 855 to 2,301 ms. Sentences were presented in quiet.

For each trial, a single sentence was randomly selected from the corpus of 336 to be in both intervals (i.e., the same sentence was presented twice). Gains were calculated for each participant’s BE4FA ear using the NAL-R formula (Byrne & Dillon, 1986; Dillon, 2012). The NAL-R formula prescribes adequate gains for linear adjustments that are comparable to the gains prescribed by nonlinear formulae at the presentation levels used in this study (Byrne, Dillon, Ching, Katsch, & Keidser, 2001; Dillon, 2012). Individual prescription gains were applied to the spectrum of all stimuli in six bands: a low-pass band with a center frequency of 0.25 kHz; four octave bands centered at 0.5, 1, 2, and 4 kHz; and a high-pass band with a center frequency of 6 kHz. Figure 1 shows the median NAL-R prescription gains across all 41 participants and key characteristics of the gain distributions.

**Figure 2.**
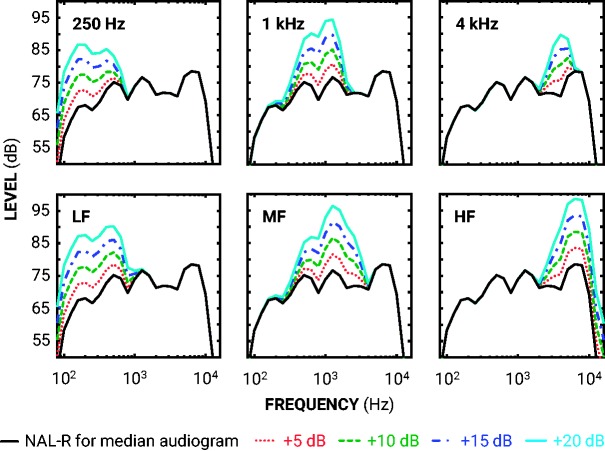
Spectra for frequency-specific gain increments. Each panel demonstrates the filter output (averaged across sentences) for each frequency-specific increment (5–20 dB; see legend for line specification) from the NAL-R standard for the median audiogram (solid black line).

Standard signals were sentences plus prescribed gains. Target signals were sentences plus prescribed gains (as with the standard signals), plus a band-specific level increment of 5, 10, 15, or 20 dB (see Figure 2). There were a total of seven conditions based on increments to the six bands mentioned earlier. There were three octave-band conditions at 0.25 (low pass), 1, and 4 kHz. There were three dual-octave-band (hereafter dual band) conditions: low frequency (LF) combining the 250 (low-pass) and 500 Hz bands, mid frequency (MF) combining the 1 and 2 kHz octave bands, and high frequency (HF) combining the 4 and 6 kHz (high-pass) bands. There was also a broadband condition, in which target signals were sentences plus prescribed gains, plus a level increment of 1, 2, 3, or 4 dB across all frequency bands. Signals were generated by convolving sentences with a 140-tap finite impulse response filter developed by Kates and Arehart (2010) for NAL-R equalization. Each filter was designed using the fir2 function in MATLAB (version 9.0.0, The Mathworks, Inc., Natick, MA), which interpolates the desired frequency response before transforming into the time domain and windowing. The filtering, however, was not optimized for the sampling frequency (44.1 kHz). This filter-sampling frequency mismatch resulted in lower- and middle-frequency filters having broader skirts than intended (see Figure 2). For a +20 dB change in gain, for example, the upper slopes of the 250-Hz and LF filters were –9 and –12 dB/octave, respectively, and the lower slopes of the 1-kHz and MF filters were both +9 dB/octave.

Standard stimuli were calibrated (using a Bruel & Kjaer Artificial Ear 4152 and Sound Level Meter 2260, Nærum, Denmark) so that with a gain of 0 dB in each band, the long-term average root mean square (RMS) presentation level across all sentences was 60 dB(A). Target stimuli were also calibrated to confirm the frequency-band and broadband increments. Audibility of the sentences was checked with the participant following their practice trials by one of the authors.

The two stimuli in each trial were separated by a silent interstimulus interval of 375 ms. Presentation level was roved from trial to trial by a randomized amount selected from a flat distribution of ± 3 dB to minimize the potential learning of a fixed-level standard. There were four possible stimulus combinations for each trial: two same (standard–standard or target–target) and two different (standard–target or target–standard). These combinations were counterbalanced and presented randomly.

### Procedure

The experiment was conducted in a single, one-and-a-half-hour session for each participant. A screening questionnaire on hearing-aid usage was completed and unmasked pure-tone thresholds were measured. Participants were seated in an audiometric booth for the experiment. Stimuli were presented monaurally over circumaural headphones (AKG K702, Vienna, Austria) after being converted from a 44.1 kHz, 16-bit digital signal to analog via an external soundcard (HRT, California, USA). Participants completed two blocks of trials with a break between, with each block lasting approximately 20 to 25 minutes. Twenty practice trials were embedded into the start of each block.

A fixed-level (method of constant stimuli), same-different task was used. Participants were asked to listen to each presentation and decide “did the sentences sound the same or different?” Participants responded by choosing the appropriate button (“same” or “different”) on a touch-screen monitor. Participants were not informed of a dimension on which to base their judgments, and therefore discrimination could be based on multiple dimensions (e.g., loudness and sound quality). Visual feedback (“correct” or “incorrect”) was provided following each response.

Twenty-eight adjustments were presented: four-level increments (5–20 dB) for each of the six frequency-band conditions (0.25, 1, and 4 kHz octave-band and LF, MF, and HF dual-band conditions) and four-level increments (1–4 dB) for the broadband (across all frequencies) condition. Coupled with four stimulus combinations (two same and two different) repeated twice, there were 224 trials (excluding practice trials) presented in a randomized order per block. Each participant completed two blocks, totaling 64 trials for each of the seven conditions.

### Analysis

Three types of JNDs were measured: (a) increment JNDs in three octave-bands, (b) increment JNDs in three dual bands, and (c) broadband increment JNDs.

Discrimination ability for each increment was calculated as *d’* (Green & Swets, 1966), assuming a same-different, differencing strategy of discrimination (Kaplan, Macmillan, & Creelman, 1978; Macmillan & Creelman, 2005). Analyses were performed with equations from Macmillan and Creelman (2005) and the Palamedes toolbox (Prins & Kingdom, 2018). Logistic functions were fit to *d’* data, with a JND corresponding to the increment at *d’* = 1. This threshold is approximately equal to 55% correct by an unbiased observer in the same-different (differencing strategy) task and 76% correct by an unbiased observer in a two-alternative forced-choice task (Macmillan & Creelman, 2005). The log–linear correction factor for extreme values was applied across the dataset (Hautus, 1995).

The Shapiro–Wilk test (Shapiro & Wilk, 1965) revealed that JNDs were not normally distributed. This was the case for octave-band JNDs (*W *=* *0.92, *p *<* *.001), dual-band JNDs (*W *=* *0.82, *p *<* *.001), and broadband JNDs (*W *=* *0.93, *p *<* *.05). Therefore, median JNDs are reported, and nonparametric analyses are employed. To avoid the influence of outliers in the JND data, Spearman rank-order correlation coefficients (ρ) are reported (Spearman, 1904). Five JNDs (approximately 2% of the total) were excluded due to poor fits resulting in extreme values.

## Results

**Figure 3.**
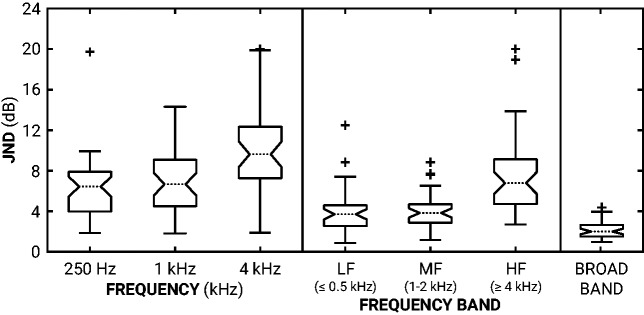
Box plot of JNDs. The left panel shows the octave-band JNDs, the middle panel shows the dual-band JNDs (LF, MF, and HF refer to low-, mid-, and high-frequency dual-octave-bands, respectively) and the right panel shows the broadband JNDs. Notches on each interquartile range (box) show the 95% confidence interval around the median. Whiskers encompass JNDs within 1.5 × interquartile range. Pluses indicate outliers with JNDs > or < 1.5 × interquartile range. JND = just-noticeable difference; LF = low frequency; MF = mid frequency; HF = shigh frequency.

Table 1 and Figure 3 show the JNDs. A Wilcoxon-signed-rank test (Wilcoxon, 1945) with the Holm-Bonferroni correction for multiple comparisons (Holm, 1979) revealed no significant differences between 0.25 and 1 kHz JNDs (*Z* = –1.41, *p *>* *.05). However, 0.25 and 1 kHz JNDs were significantly smaller (better) than 4 kHz JNDs (*Z* = –3.90 and –4.05, respectively; *p *<* *.001 for both). A similar effect of center frequency was found for the dual-band JNDs. While LF and MF JNDs were not significantly different (*Z* = –0.56, *p *>* *.05), they were smaller than the HF JNDs (*Z* = –4.23 and –4.29, respectively; *p *<* *.001 for both). Of note are the large individual differences, reflected in the interquartile ranges (see Table 1); for example, 25% of participants could not discriminate a +5 dB change in LF or MF gain.

**Table 1.** Summary of Results.

	JNDs (dB)	
Increment	Median	IQR
250 Hz	6.3	4.0–7.8
1000 Hz	6.7	4.6–9.1
4000 Hz	9.6	7.3–12.4
Low frequency	3.7	2.5–4.6
Mid frequency	3.8	2.9–4.7
High frequency	6.8	4.7–9.1
Broadband	2.0	1.5–2.7

*Note*. Median JNDs and their interquartile (25% –75%) ranges (in dB) for each condition are given. JND = just-noticeable differences; IQR = interquartile range.

Octave-band JNDs were greater (poorer) than dual-band JNDs: 0.25 kHz JNDs were greater than LF JNDs (*Z *=* *4.15, *p *<* *.001), 1 kHz JNDs were greater than MF JNDs (*Z *=* *4.44, *p *<* *.001), and 4 kHz JNDs were greater than HF JNDs (*Z *=* *3.31, *p *<* *.001). Octave-band JNDs were also greater than broadband JNDs (*Z *=* *5.24, 5.48, and 5.50, respectively, for the 0.25, 1, and 4 kHz bands; *p *<* *.001 for all), as were dual-band JNDs (*Z *=* *4.64, 5.09, and 5.57, respectively, for LF, MF, and HF bands; *p *<* *.001 for all).

Correlations between participants’ frequency-specific JNDs and their corresponding pure-tone thresholds were examined. Correlations between individual 0.25, 1, or 4-kHz JNDs and their respective 0.25, 1, or 4-kHz pure-tone thresholds were insignificant (all *p *<* *.05). For dual-band discrimination, LF and MF JNDs were not significantly correlated with corresponding average pure-tone thresholds (both *p *>* *.05), while participants’ averages of pure-tone threshold at 4 and 6 kHz were positively correlated with their HF JNDs (ρ = 0.34, *p *<* *.05). To examine audibility more directly, JNDs were also compared with sensation level, which was approximated from individual thresholds and long-term presentation level as determined by individual prescription. The same relationship was found: only dual-band HF sensation levels were correlated with HF JNDs (ρ = –0.50; *p *<* *.001, respectively). There were no significant correlations between JNDs and age or hearing-aid experience when adjusting for multiple comparisons with the Holm-Bonferonni correction (all *p *>* *.05).

**Figure 4.**
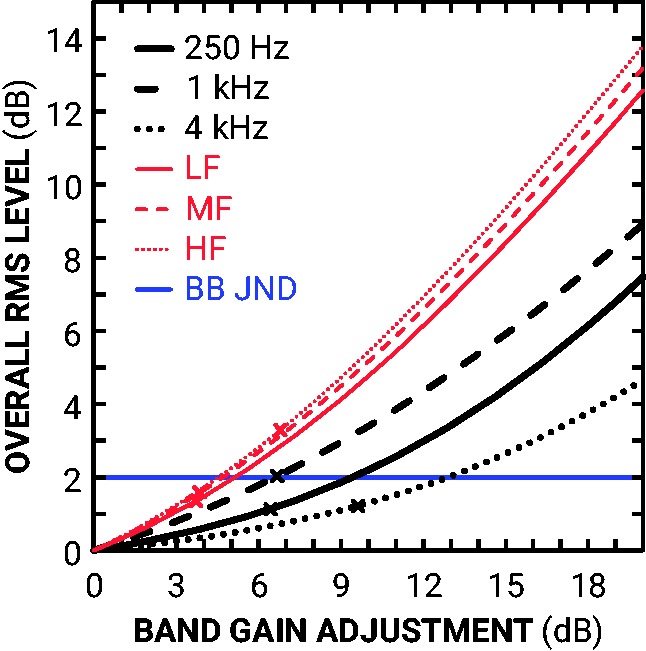
Line plot showing the effect of frequency-band gain increments on overall root mean square (RMS) level. The blue horizontal line references the broadband JND. The thick black and thin red lines refer to overall RMS levels as a function of octave- and dual-band increments, respectively, at low (solid), middle (dashed), and high (dotted) center frequencies. RMS levels were averaged across the BKB corpus. The crosses on these lines refer to the median JND for that particular increment. JND = just-noticeable difference; BKB = Bamford–Kowal–Bench.

Given the magnitude of the frequency-specific JNDs, it is possible that the increments in frequency-specific gain were affecting the overall level beyond the broadband JND. Judgments may have been based on overall level cues rather than the frequency-specific increments. To examine this possibility, the overall RMS level change for gain increments in each of the six bands was calculated using the entire corpus of BKB sentences (see Figure 4). Incrementing by the median JND for the 0.25 and 4 kHz, LF and MF bands produced overall RMS level changes below the broadband JND (2.0 dB). For the 1 kHz band however, a gain increment of one median JND resulted in an overall level change similar to the broadband JND. In addition, a gain increment of one median JND to the HF band resulted in an overall level change 1.2 dB greater than the broadband JND.

## Discussion

This study measured the JNDs for level increments in male, single-talker sentences, presented with prescribed gains in quiet to the BEs of hearing-impaired listeners. The results are summarized in Table 1 and Figure 3. JNDs were both center frequency and bandwidth dependent. In terms of center frequency, higher frequency JNDs were significantly greater (poorer) than the lower frequency JNDs for both bandwidths of adjustment. This disparity may have occurred due to the relatively low high-frequency energy in the sentences. In terms of bandwidth, octave-band JNDs were significantly greater than corresponding dual-band JNDs, and all dual-band JNDs were significantly greater than broadband JNDs. This is in line with previous profile analysis research that increment detection improves with bandwidth (Bernstein & Green, 1987). However, 0.25 kHz octave-band JNDs were smaller (*Z* = –2.19, *p *<* *.05) than HF dual-band JNDs, indicating discrimination ability is more dependent on the energy within a band than bandwidth. With the scale of these octave- and dual-band JNDs, it was possible that participants were judging changes in overall loudness rather than spectral shape. For most octave-band and dual-band adjustments, however, a gain increment of one JND did not exceed the broadband JND (see Figure 4). These findings suggest that participants’ judgments were largely based on the changes in spectral shape resulting from the increments within bands rather than on overall loudness cues.

As shown in Table 2, the current frequency-specific JNDs were highly elevated compared with those measured in Caswell-Midwinter and Whitmer (2019) with speech-shaped noises. As in previous research (Whitmer & Akeroyd, 2011), the broadband JND for speech-shaped noises was lower (better) than the broadband JND for sentences. The similarity in broadband JNDs but wide differences in octave-band JNDs between speech and noise stimuli suggest that the spectro-temporal complexity of the speech is more likely to be a significant mediator of the octave-band speech JNDs than duration. Apart from the 0.25 kHz frequency band, speech-shaped noise JNDs were broadly center frequency independent. In this study, the high-frequency octave-band and dual-band JNDs were significantly greater than others. The speech used here had an unnaturally steep low-pass cut-off at 10 kHz (see Figure 2) compared with the speech–spectrum noise. Each speech-shaped noise interval was relatively steady over 500 ms, while the speech had wide envelope fluctuations, which may further explain the disparity between speech-shaped noise and speech JNDs. Another source of disparity between speech-shaped noise and speech JNDs may have been the trial-by-trial stimulus variations. Speech-shaped noises with similar spectral profiles were presented across trials in the previous study, while in this study, randomly selected sentences were presented across trials. It could also be expected that the current task required greater informational processing compared with the previous task with speech-shaped noises, given that the speech conveyed linguistic meaning. This may have caused further disparity between speech and speech-shaped noise JNDs.

**Table 2.** Current Speech JNDs Compared With Speech-Shaped Noise JNDs.

	JNDs (dB)	
Increment	Speech	Noise
250 Hz	6.3	4.5
1000 Hz	6.7	2.4
4000 Hz	9.6	2.8
Broadband	2.0	1.5

*Note*. Median JNDs in dB for sentences and speech-shaped noises (Caswell-Midwinter & Whitmer 2019) for octave-band and broadband conditions. JND = just-noticeable differences.

There was a positive correlation between HF JNDs and averaged pure-tone thresholds at 4 and 6 kHz (ρ = 0.34). Further analyses revealed that the two participants with HF JNDs of 18 dB or greater had averaged 4 and 6 kHz pure-tone thresholds of at least 75 dB HL. There was also a negative correlation between HF JNDs and sensation level at 4 and 6 kHz (ρ = –0.50). These correlations suggest an effect of audibility for this condition. The sensation levels in these higher frequency bands were not sufficient for all participants to make accurate judgments on lesser increments, resulting in elevated JNDs. Caswell-Midwinter and Whitmer (2019) similarly reported a positive correlation between 6 kHz pure-tone thresholds and 6 kHz speech-shaped noise JNDs (*r *=* *0.39), which was driven by participants with the most severe 6 kHz pure-tone thresholds. These findings suggest that patients will require even larger adjustments than the median JNDs to be noticeable if they are not provided with adequate sensation levels. As in Caswell-Midwinter and Whitmer (2019), there was no evidence of a relationship between JNDs and hearing-aid use.

The 2.0 dB broadband JND measured in this study is commensurate with the unaided and aided broadband JNDs for sentences in Whitmer and Akeroyd (2011). Despite some differences in tasks, both used level roving across trials to minimize learning of the standard stimulus. As hearing-aid fittings often involve live voice for gain verification (Dillon, 2012; Jenstad et al., 2003; Thielemans et al., 2017), level fluctuations across sentences would be expected in both clinical and less-controlled self-fittings. Hence, while lower JNDs could be expected without roving due to learning, they would not be more ecologically valid.

Measuring intelligibility and sound quality ratings with hearing aids, Dirks et al. (1993) and Jenstad et al. (2007) inferred a 4.5 dB broadband JND for speech. This suggests that the gain adjustments required to meet changes in subjective dimensions are likely to be larger than those that are discriminable. The current method used a dimensionless same-different task to measure the discrimination ability of an acoustical adjustment, irrespective of the internal criteria underlying a participant’s judgment. It would, however, also be of value to measure the frequency-specific adjustments away from prescription in a more qualitative task. Such a task could clarify the smallest adjustments required to change subjective judgments such as those on preference or sound quality, which are common in fine-tuning and troubleshooting. It should be noted that compared with current hearing aids with multiple frequency bands per octave, this study limited the frequency specificity of adjustments to octave and dual-octave bands with broad skirts in the lower and middle frequencies (see Figure 2). The JNDs for narrower band adjustments would be expected to be even greater than those measured here (cf. Bernstein & Green, 1987).

This study presented recorded male, single-talker sentences from the BKB corpus. This material is standardized, providing a starting point for examining the discriminability of speech adjustments. However, there is some variation in spectral content between talkers: these JNDs may be over or underestimates depending on speech characteristics such as language or gender (Byrne, Dillon, & Tran, 1994). Accent and familiarity that have been shown to affect speech intelligibility may also affect JNDs (Adank, Evans, Stuart-Smith, & Scott, 2009; Smith, Holmes-Elliott, Pettinato, & Knight, 2014). This study also presented sentences from a relatively large corpus. Future study presenting a smaller set of sentences or words with defined phonetic and spectral characteristics (e.g., speech containing a high proportion of high-frequency alveolar plosives) may improve the discriminability of frequency-specific gain adjustments.

Given the sparseness of information in any frequency band of speech at any time in these short sentences (mean duration 1.6 s), it is possible that longer duration speech—such as an ongoing monologue—may improve JNDs (cf. Dai & Green, 1993; Shrivastav, Humes, & Kewley-Port, 2006). A more clinically efficient speech stimulus could be concatenated phonemes targeting particular frequency regions (e.g., “babababa”). It is also possible that speech, given its fluctuations in any given frequency band, is not the appropriate stimulus for the personalization of gain. Using different sounds such as music with separable low-, middle-, and high-frequency content (e.g., a jazz piano trio) could possibly yield lower JNDs, although these may not be the preferred gains for speech signals.

Judgments were made on a short-term, trial-by-trial basis, similar to those made in a hearing-aid fitting. However, it is not known whether the JNDs change with time. It may be that sensitivity improves with listening experience; that a small adjustment previously unnoticeable in the clinic becomes noticeable and appreciable after a period of real-world use. In this case, these results may still be of use to a clinician to inform a patient in an appointment that while instant benefits from fine-tuning are unlikely, they may be appreciable with time. Alternatively, it may be that sensitivity worsens with time. This study was conducted in favorable listening conditions, and it is reasonable to expect that noisier environments would degrade sensitivity. Further research on the longitudinal, real-world effects of JND-sized adjustments on subjective and objective outcomes is required. Earlier studies have disagreed on whether JND-sized deviations from prescriptions would be detrimental (Boymans & Dreschler, 2012) or not (Van Buuren, Festen, & Plomp, 1995). Recent evidence suggests that self-adjusted gains can maintain speech intelligibility in quiet and in noise, even if deviating widely from prescription (Nelson et al., 2018). The step sizes in gain suggested by these results should be currently considered only in terms of what is noticeable to patients, whether fitting by themselves or by a clinician.

## Conclusions

Frequency-dependent gain is routinely adjusted to patient feedback in hearing-aid fittings. Speech is a common test signal for these adjustments, particularly in fine-tuning and troubleshooting practices. The JNDs reported here (see Table 1) provide evidence for noticeable gain adjustments made to speech. A noticeable frequency-specific gain adjustment should be several times larger (4–10 dB dependent on center frequency and bandwidth) than an overall gain adjustment (2 dB). High-frequency dual-octave-band JNDs were positively correlated with high-frequency pure-tone thresholds and sensation levels; this suggests that patients will require even larger adjustments to be noticeable if they are not provided with adequate sensation levels. For all other conditions tested, there was no evidence that degree of hearing loss affected JNDs. The magnitude of these JNDs suggest that short sentences may not be the most efficient signal for adjusting hearing-aid gain to patient feedback. Future studies should explore how the discriminability of gain adjustments affects the qualitative judgments involved in hearing-aid fittings.
